# Origin of superior thyroid artery: under the surgeon’s knife

**DOI:** 10.1590/1677-5449.004218

**Published:** 2018

**Authors:** Ranjith Sreedharan, Lalu Krishna, Ashwija Shetty

**Affiliations:** 1 Jubilee Mission Medical College and Research Institute, Department of Anatomy, Thrissur, Kerala, India.; 2 Kasturba Medical College, Department of Anatomy, Manipal, Karnataka, India.

**Keywords:** external carotid artery, superior thyroid artery, superior laryngeal nerve, anatomic variation, artéria carótida externa, artéria tireoidea superior, nervo laríngeo superior, variação anatômica

## Abstract

**Background:**

The major arterial supply to the thyroid gland is from the superior and inferior thyroid arteries, arising from the external carotid artery and the thyrocervical trunk respectively. The external laryngeal nerve runs in close proximity to the origin of the superior thyroid artery in relation to the thyroid gland. The superior thyroid artery is clinically important in head and neck surgeries.

**Objectives:**

To locate the origin of the superior thyroid artery, because wide variability is reported. To provide knowledge of possible variations in its origin, because it is important for surgical procedures in the neck.

**Methods:**

The origin of the superior thyroid artery was studied by dissecting sixty adult human hemineck specimens from donated cadavers in a Department of Anatomy.

**Results:**

The highest incidence observed was origin of the superior thyroid artery from the external carotid artery (88.33%), whereas origin from the common carotid bifurcation only occurred in 8.33%. However, in 3.33% of cases, the superior thyroid artery originated from the common carotid artery and in a single case, the external laryngeal nerve did not cross the stem of the superior thyroid artery at all, but ran ventral and parallel to the artery.

**Conclusions:**

It is important to rule out anomalous origin of superior thyroid artery and verify its relationship to the external laryngeal nerve prior to ligation of the artery in thyroid surgeries, in order to prevent iatrogenic injuries. Moreover, because anomalous origins of the superior thyroid artery are only anatomic variants, thorough knowledge of these is decisive for head and neck surgeries.

## Resumo

**Contexto:**

O suprimento arterial principal para a glândula tireoide provém das artérias tireoideas superior e inferior, que têm origem na artéria carótida externa e no tronco tireocervical, respectivamente. O nervo laríngeo externo faz um percurso bem próximo à origem da artéria tireoidea superior em relação à glândula tireoide. A artéria tireoidea superior é clinicamente importante em cirurgias da cabeça e do pescoço.

**Objetivos:**

Localizar a origem da artéria tireoidea superior, considerando a ampla variabilidade descrita na literatura; e oferecer informações sobre possíveis variações em sua origem, devido à importância disso para procedimentos cirúrgicos realizados no pescoço.

**Métodos:**

A origem da artéria tireoidea superior foi estudada dissecando-se 60 espécimes de hemipescoço adulto de cadáveres humanos doados ao Departamento de Anatomia.

**Resultados:**

A maior incidência observada foi da artéria tireoidea superior com origem na artéria carótida externa (88,33%), enquanto a origem na bifurcação da artéria carótida comum ocorreu em apenas 8,33%. No entanto, em 3,33% dos casos, a artéria tireoidea superior teve origem na artéria carótida comum, e em um único caso, o nervo laríngeo externo não cruzou o tronco da artéria tireoidea superior em nenhum momento, embora tenha cursado ventral e paralelamente a essa artéria.

**Conclusões:**

É importante descartar origem anômala da artéria tireoidea superior e confirmar sua relação com o nervo laríngeo externo antes da ligadura da artéria em cirurgias da tireoide, para evitar efeitos iatrogênicos. Além disso, como origens anômalas da artéria tireoidea superior são apenas variantes anatômicas, o conhecimento detalhado dessas variações é decisivo para cirurgias da cabeça e do pescoço.

## INTRODUCTION

 The superior thyroid artery (STA) is considered to have a fairly persistent origin from the ventral surface of the external carotid artery (ECA), just inferior to the greater cornu of the hyoid bone, terminating in the thyroid gland. One of its major branches is the superior laryngeal artery (SLA). It supplies the adjacent muscles, the upper larynx, and the neck region; and it gives off innumerable branches to the thyroid gland and overlying skin. 1 ^,^ 2 Conflicting reports have been published regarding the origins of the STA and SLA, such as the former arising from the common carotid artery (CCA) (1.5-47% of cases) or at the point of bifurcation of the common carotid artery (CCB) (21-49% of cases), and the latter arising from the ECA. 3 ^-^ 8 Anatomical variations of the STA are important during surgical and radiological intervention in the neck. Surgical procedures such as radical neck dissection, thyroidectomy, reconstruction of an aneurysm, cricothyroidotomy, carotid endarterectomy, cancer therapies, interventional radiology and plastic surgery all involve the STA. 4 ^,^ 5 ^,^ 9 Angiologists and head and neck surgeons should be familiar with variations in the origin and course of the principal arteries of the head and neck during surgical procedures and treatment of the upper larynx, parathyroid and thyroid glands. 10 Esen et al. 11 advise conducting a thorough CT angiograph study to understand the arterial system of the head and neck region prior to any surgical approach. Thyroidectomy is the most common head and neck surgery done worldwide and the mortality rate is considered to be 0%. Although the rate of complications is lower than 3% (including compressive hematoma, recurrent laryngeal nerve palsy and hypoparathyroidism) they are highly significant in medico-legal cases. 12 All surgeries run the risk of complications which can be fatal and therefore risks must be analyzed and minimized in all situations. This study aimed to evaluate the frequency of usual anatomical variations in the origin of the STA in human cadavers and compared them with the results obtained in earlier studies. 

## MATERIALS AND METHODS

 The origin of the STA was studied in sixty adult human hemineck specimens, irrespective of sex. The specimens were collected from donated cadavers of the same ethnic group, which were used during routine dissection by medical undergraduates in a Department of Anatomy. A detailed observational study was conducted on the origin of the STA in the anterior triangle in these 60 hemineck specimens over a period of 5 years. Of these, 42 hemineck specimens were from the same cadaver (21 head and neck specimens) and 18 heminecks were right and left sides from different cadavers. Clinical histories were available, and none of the cases showed any reference to vascular interventions around the neck. When variations in the origin of the STA were identified, they were explored further, numbered and photographed. The results reported in earlier studies were reviewed and compared with the results of the present study. 

## RESULTS

**Figure 1:**
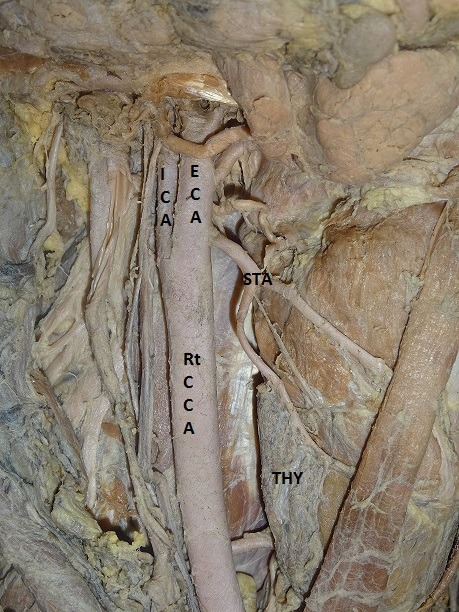
Origin of superior thyroid artery from external carotid artery. This represents the most common pattern of origin of the superior thyroid artery. Rt CCA = right common carotid artery; ECA = external carotid artery; ICA = internal carotid artery; STA = left superior thyroid artery; THY = thyroid gland.

**Figure 2:**
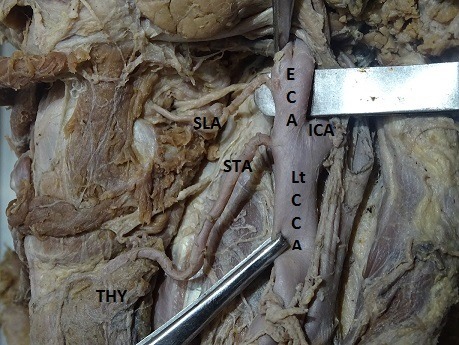
Origin of left superior thyroid artery from common carotid bifurcation. Lt CCA = left common carotid artery; ECA = external carotid artery; ICA = internal carotid artery; SLA = superior laryngeal artery; STA = left superior thyroid artery; THY = thyroid gland.

**Figure 3:**
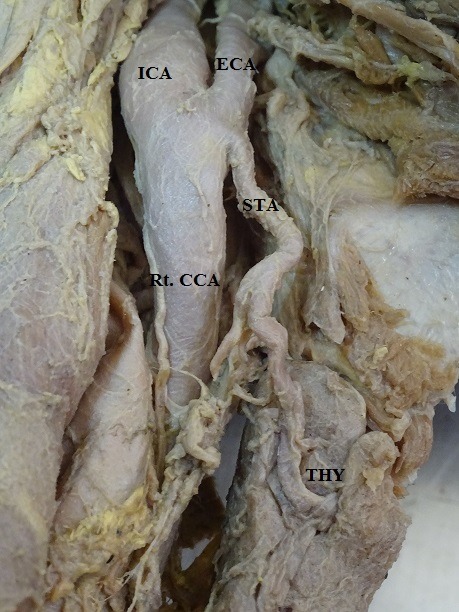
Origin of right superior thyroid artery from common carotid bifurcation. Rt CCA = right common carotid artery; ECA = external carotid artery; ICA = internal carotid artery; STA = right superior thyroid artery; THY = thyroid gland.

**Figure 4:**
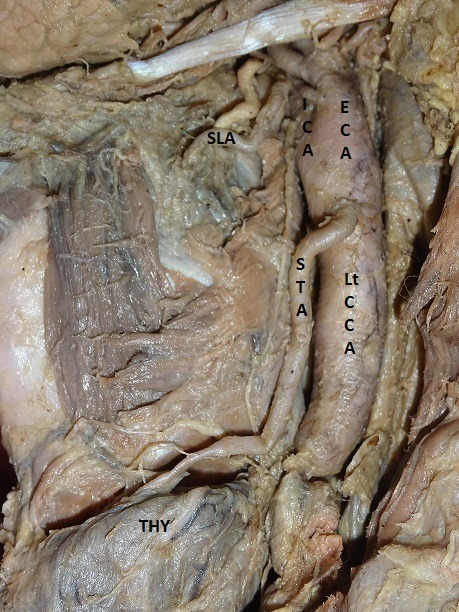
Origin of left superior thyroid artery from left common carotid artery. Lt CCA = left common carotid artery; ECA = external carotid artery; ICA = internal carotid artery; STA = left superior thyroid artery; THY = thyroid gland.

**Figure 5:**
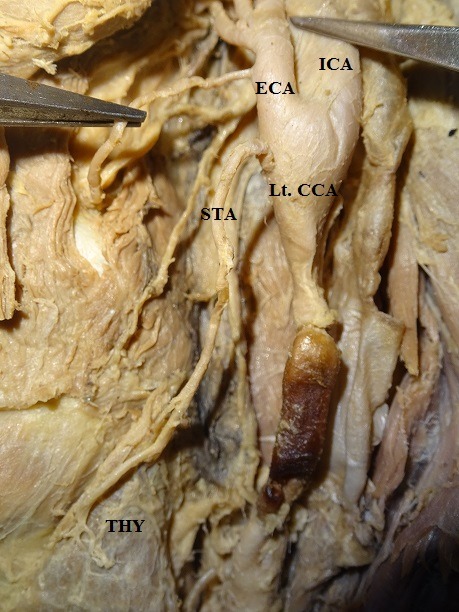
Origin of left superior thyroid artery from left common carotid artery. External laryngeal nerve did not cross the stem of superior thyroid artery at all, but ran ventral and parallel to the artery. Lt CCA = left common carotid artery; ECA = external carotid artery; ICA = internal carotid artery; STA = left superior thyroid artery; THY = thyroid gland.

 The results showed that the STA originates from the ECA, CCB, or CCA, with different frequencies ( Table 1 ). In 53 of the 60 hemineck specimens, the STA arose from ECA, with an incidence of 88.33% ( Figure 1 ). The site of origin of the STA showed no statistically significant difference by side (p > 0.05) and frequencies of occurrence were 50.94% on the right and 49.06% on the left. In 8.33%, the STA arose at the CCB, which was observed in 5 of the 60 heminecks. Three (60%) of these 5 cases were right side and 2 (40%) left side heminecks ( Figures 2 and 3 ). In 2 of the 60 cases, with an incidence of 3.33%, the STA arose from the CCA. This was infrequent and in both cases it arose from the left CCA ( Figure 4 ). Moreover, in these two cases, the SLA arose from the ECA and the external laryngeal nerve (ELN) did not cross the stem of the STA at all, but ran ventral and parallel to the artery ( Figures 4 and 5 ). A bilateral variation was observed in only 1 of the 21 head and neck specimens (in which the specimen had both right and left heminecks). Here, the STA arose from the CCB on the left side and from the ECA on the right side. In right and left sides combined, in 96.66% of cases, the level of the origin of STA was above the level of the superior border of the thyroid cartilage, in 1.66% the origin was at the level of the upper border of cartilage, and in 1.66% the origin was below the upper border ( Table 2 ). 

**Table 1:** Site of origin of the STA.

**Site of origin of the STA**	**Right side** **(n = 30) (%)**	**Left side** **(n = 30) (%)**	**Total** **(n = 60) (%)**
External carotid artery	27 (90)	26 (86.66)	53 (88.33)
Common carotid bifurcation	03 (10)	02 (6.66)	05 (8.33)
Common carotid artery	00 (0)	02(6.66)	02 (3.33)

STA = superior thyroid artery.

**Table 2:** Level of origin of the STA in relation to the upper border of the thyroid cartilage.

**Level of origin of STA**	**Right side** **(n = 30) (%)**	**Left side** **(n = 30) (%)**	**Total** **(n = 60) (%)**
Above upper border of thyroid cartilage	30 (100)	28 (93.33)	58 (96.66)
Upper border of thyroid cartilage	00 (0)	01 (3.33)	01 (1.66)
Below upper border of thyroid cartilage	00 (0)	01 (3.33)	01 (1.66)

STA = superior thyroid artery.

## DISCUSSION

 Dessie 13 conducted a study with 86 specimens and reported that the STA originated from the ECA in 44.2% of cases, from the CCA in 27.9% of cases, and from the CCB in 26.7%. Gupta et al. performed twenty-five angiographic studies of 15 patients and identified the origins of the STA. In their study, the STAs on the right side of the body arose from the ECA, CCB, and CCA and, in one case, from the internal carotid artery (ICA), at proportions of 71.5%, 21.5%, 7%, and 7% respectively. 9 On the left side 72.5% of STA arose from the ECA, 18.5% from the CCB, and 9% from the ICA. Studies of the origin of the STA conducted by Lucev et al., 3 Vázquez et al., 4 Ozgur et al., 5 Natsis et al., 14 Joshi et al. 6 and Shivaleela et al. 7 found that it arose from the ECA, CCB, or CCA. The proportions reported were compared with those observed in the present study and tabulated ( Table 3 ). A study of 100 carotid arteries reported that the STA originated from the ECA in 39% and from the level of the carotid bifurcation and the CCA in 61% of cases. 14 In the present study, a higher incidence (88.33%) of origin of the STA from the ECA was noted, while origin from the CCB only accounted for 8.33% of cases. These observations are not closely aligned with those of previous studies. However, in 3.33% of cases, the superior thyroid artery originated from the CCA. This observation is closely aligned with studies on Indian populations by Joshi et al. 6 and Shivaleela et al. 7 Both Toni and Ongeti suggested that ethnic differences were the cause of discrepancy in the origin of STA in comparison with other populations and only Eid suggested transformation of the aortic arches. 15 ^-^ 17 Other than the STA, the lingual artery has also been reported to have anomalous origins from the CCA and its bifurcation. 18 Formation of branches of the ECA includes complicated processes such as annexation, regression and remodelling. 19 Angiogenesis theory suggests that confluence of the vessels and vessels with large diameter are more common in fetuses compared with adults. 20 Any variation in this process of angiogenesis could lead to the unusual ECA branching pattern. In general, the SLA arises from the STA. It is reported that the SLA can arise from the ECA, but none of these studies mentioned the origin of the SLA. 21 ^-^ 23 In the present study, the SLA arose from the ECA in cases in which the STA took its origin from the CCA. Among variations in the origin of the STA, the most common relationship to the superior border of the thyroid cartilage was at the level above the superior border, in 96.66% of cases, at the level of the upper border in 1.66% of cases and at the level below the superior border in 1.66% of cases, right and left sides combined. According to Shivaleela et al., 7 in 88.09% of cases, the STA arose above the level of the superior border of the thyroid cartilage, whereas in 11.90% of cases it arose at the level of the superior border. Moreover, an interesting observation was made with regard to the relationship between the STA and the ELN in one out of two cases in which the STA arose from the left CCA, 1 cm proximal of the point of bifurcation. In this case, the ELN did not cross the stem of the STA at all, but ran ventral and parallel to the artery until it had ramified. This unique relationship differs from the types classified by Kierner et al. 24 Therefore, care must be taken to identify and preserve the ELA during neck surgeries, since the ELN supplies the cricothyroid muscle and keeps the vocal folds under tension. Unilateral injury of the ELN leads to mild voice huskiness; while bilateral injury often results in much more devastating outcomes. 

**Table 3:** Origin of superior thyroid artery based on incidence.

**Author and year of publishing**	**No. of cases/total sample (%)**		
	**ECA**	**CCB**	**CCA**
Lucev et al. 3	12/40 (30)	9/40 (22)	19/40 (47)
Vázquez et al. 4	48/207 (23)	102/207 (49)	55/207 (26.6)
Ozgur et al. 5	5/20 (25)	8/20 (40)	7/20 (35)
Joshi et al. 6	44/66 (66.67)	21/66 (31.81)	01/66 (1.51)
Shivaleela et al. 7	64/84 (76.19)	18/84 (21.43)	2/84 (2.38)
Won 8	6/30 (20)	12/30 (40)	12/30 (40)
Dessie 13	38/86 (44.2)	24/86 (27.9)	23/86(26.7)
Present study	53/60 (88.33)	5/60 (8.33)	2/60 (3.33)

CCA = common carotid artery; CCB = bifurcation of carotid artery; ECA = external carotid artery.

## CONCLUSION

 Current advances in surgical practices have created a need for thorough appreciation of common and unusual anatomical aberrations possible in a given region. If the STA is not found in its usual position with respect to its origin, its relation to the ELN and thyroid cartilage must be considered. Awareness of the findings of this study will be helpful in surgical procedures such as radical neck dissection, thyroidectomy, aneurysm reconstruction, cricothyroidoctomy, carotid endarterectomy, cancer therapies, interventional radiology, and plastic surgery. To summarize, anomalous origins of the STA are only anatomic variants, but thorough knowledge of them helps to prevent iatrogenic injuries. 
